# Supplementary material for: Macmoondongtang modulates Th1-/Th2-related cytokines and alleviates asthma in a murine model
Source: PLoS One. 2019 Dec 2;14(12):e0224517. doi: 10.1371/journal.pone.0224517 (PMC6886797; doi:10.1371/journal.pone.0224517)
Supplement: Attachment — Submitted filename: Response to Reviewers comment.docx [file pone.0224517.s004.docx]

Reviewer #2:

1. My comment 2 is not addressed in the revised manuscript. Please change the conlusions on page 3. I would suggest to modify the conclusions to: “Macmoondongtang treatment alleviates asthma symptoms and modulate the Th1-/Th2- related cytokines. Glycyrrhizin and liquiritin could be the major the active therapeutic components”.

Ans) Thank you so much for your suggestion and I amended the conclusion part in the abstract.

Reviewer #1: Major comments:
After revise, the manuscript has been improved by the authors. But two major things still remain to be improved, as follows:
1, the writing must be improved. 1) introduction, special paragraph 2 and 3, is not so clear to this study; 2) discussion, which has been improved is not enough. Furthermore, some description is misleading, such as the synergistic role between glycyrrhizin and liquiritin unless there are some experimental finding available.
2, the microscropic figures are in a low quality. My previous comment has mentioned this problem. The authors' response was that it had been adjusted. But there are not clear, I don't know why.

Minor comments:
1, there are still some grammar mistakes to be checked.
2, ref #8, is not completed.

Reviewer #2: In this revised manuscript, the authors report that macmoondongtang could regulate host immune response in a murine model of asthma. Specifically, mice treated with macmoondongtang show decreased neutrophil count in BALF, reduced T-bet, IFN-g, and TNF-a, IL-12, IL-4, and IL-5 genes expression in the lung. While most of my previous concerns have been addressed, the reviewer still has some minor comments about several conclusions in the manuscript. The authors provide no data supports that modulation of Th1/Th2 cytokines is the mechanism of how Macmoondongtang alleviates asthma.
1. I would suggest change the title to “Macmoondongtang modulates Th1-/Th2-related cytokines and alleviates asthma in a murine model”.
2. I would suggest modify the conclusions to: “Macmoondongtang treatment alleviates asthma symptoms and modulate the Th1-/Th2- related cytokines. Glycyrrhizin and liquiritin could be the major the active therapeutic components”.
