# Supplementary material for: Macmoondongtang modulates Th1-/Th2-related cytokines and alleviates asthma in a murine model
Source: PLoS One. 2019 Dec 2;14(12):e0224517. doi: 10.1371/journal.pone.0224517 (PMC6886797; doi:10.1371/journal.pone.0224517)
Supplement: Attachment — Submitted filename: Replies to Reviewer comments.docx [file pone.0224517.s002.docx]

Please use the space provided to explain your answers to the questions above. You may also include additional comments for the author, including concerns about dual publication, research ethics, or publication ethics. (Please upload your review as an attachment if it exceeds 20,000 characters)

**Reviewer #1**

Comment #1

The manuscript entitled with “Macmoondongtang modulates asthma-related changes via modulation of TNF-a and T-bet & IFN-r in an asthma murine model” brought some interesting findings. However, it is not convincible for its description and conclusion with several reasons as follows:

1) Macmoondongtang has been used as a traditional prescription for pulmonary disease in Korea. However, there is no report about the mechanism involved in its effect. In the present study, the authors described how Macmoondongtang as a repiratory medicine has effect on Ashma in a murine model. Since Macmoondongtang is a traditional prescription for pulmonary disease, surprisingly, there is no any information on how Macmoondongtang has been clinically used in Ashma treatment at all. So, it is really weird for me to choose Ashma instead of the other disease models for study on Macmoondongtang.

ans) Thank you for your kind advise.

Macmoondongtang has been used as oriental medicine in Korea and China and is still commercialized. Macmoondongtang was generally used to cure bronchitis, asthma, pneumonia, cough, and sputum production. The mechanism of anti-asthmatic activity of Macmoondongtang has not been reported. Therefore, we have demonstrated the anti-asthmatic effect of Macmoondongtangin animal experiments used commercial products (see Materials and methods). The effective dose of Macmoondongtang in animal studies was determined to be 1500 mg/kg. We calculated the dose of human based on the results of animal experiments. Human dose was included in the daily intake range of commercial products (see, discussion section). The important facts in this manuscript are that anti-asthmatic effect has been demonstrated within the stable dose range of Macmoondongtang, and the action mechanism has also been clearly identified.

2) According to the results, it looks like that Macmoondongtang play role in modulation of expression of Th1, Th2 and Th17-related cytokines. Why did the manuscript choose a tittle as modulation of TNF-a and T-bet & IFN-r? It doesn't make any sense to me.

Ans) Thank you so much for your comments and I agreed with your comments. I think that the manuscript title should be changed to “Macmoondongtang controls the asthmatic changes via modulation of Th1-/Th2-/Th17-related factors in an asthma murine model”.

3) The results showed that Macmoondongtang contained glycyrrhizin and liquiritin. Theoretically glycyrrhizin and liquiritin could have some role in modulation of the cytokine expression. But how did you exclude effects of some unknown components in Macmoondongtang.

ans) Thank you for your kind advise. The effective dose of glycyrrhizin was reported to be 2.5-20 mg/kg in the mouse model (Ram et al) and liquiritin has been reported to be efficacious at a minimum dose of 5 mg/kg in mouse model (Ram et al). We reported that in the discussion section that Macmoondongtang contained 0.38% glycyrrhizin. Glycyrrhizin in Macmoondongtang was calculated based on the content and correlated with animal experimental results. The dose of 1500 mg/kg Macmoondongtang is equivalent to 5.7 mg/kg glycyrrhizin. These results are correlated with previous reports (efficacy range 2.5 - 20 mg/kg). It can be interpreted that glycyrrhizin present in Macmoondongtang shows the main anti-asthmatic effect. Based on the content of liquiritin, the effective dose (5 mg/kg or more) is insufficient. Therefore, liquiritin was thought to be a cofactor for anti-asthmatic effect.

Comment #2

Has the statistical analysis been performed appropriately and rigorously? No Although the manuscript showed thirty-fine mice were divided into five groups, it was not clear of how many mice had being used in each figure. So, it is very confused about how they calculated the data without showing “n” in each figure.

Ans) Thank you so much for your advices. This study was conducted twice time using with 70 heads, for one study 35 mice had been used and using with same method the other study was done. I added this method in the Materials and Methods section and used numbers of animals for each graph was described in the Figure Legends.

Comment #3

Is the manuscript presented in an intelligible fashion and written in standard English? No

There are a lot of places to be rewrote in a standard English in this manuscript, for example, ”It is important to decrease IgE expression increased by ovalbumin treatment”, “T-bet is a factor for Th1 cell transcription…., to the end of this paraph”, and so on.

Ans) Thank you so much for your advice. Although this manuscript had been edited by professional editing company (**I attached the certificate file of editing below**) revised manuscript would be done by editing company again.

Some minor comments:

1)For material and methods, there were a lot of description to be improved, for example, which samples were used for RNA extraction? how the samples were prepared for IFA? and so on.

Ans) Thank you so much for your comments and I add the description about materials and methods in the Section.

2) What’s magnitude for those microscopic figures? And the quality of those microscopic figures should be also improved.

Ans) Thank you so much for your advice and I added the magnification in each photo. For improving the photos’ quality, I adjusted them again.

**Reviewer #2**

In this study, Lee et al. reported that macmoondongtang, a traditional prescription for treating respiratory disease, could modulate host immune responses during OVA-induced asthma in a mice model. They reported that macmoondongtang treatment could reduce serum IgE level, mucus hypersecretion, inflammatory cell infiltration in the lungs. They also reported that macmoondongtang treatment leads to reduced expression of Th1/Th2 transcriptional factors as well as related cytokines such as IFN-g, IL-12, IL-4 and IL-5, IL-13 on mRNA level or protein level. The data is interesting; however, the manuscript has severe limitations that the authors need to deal with before publications.

1. The written Englished should be revised significantly. There are many grammar mistakes in the manuscript.

Ans) Thank you so much for your advice. Although this manuscript had been edited by professional editing company (**I attached the certificate file of editing below**) revised manuscript would be done by editing company again.

2. The conclusions are overstated and need revision. For example,

a. it is not convincing that glycyrrhizin and liquiritin were the effective ingredients of macmoondongtang.

ans) Thanks for your kind advise. Macmoondongtang has been used as oriental medicine in Korea and China and is still commercialized. Macmoondongtang was generally used to cure bronchitis, asthma, pneumonia, cough, and sputum production. The mechanism of anti-asthmatic activity of Macmoondongtang has not been reported. Therefore, we have demonstrated the anti-asthmatic effect of Macmoondongtangin animal experiments used commercial products (see Materials and methods). The effective dose of Macmoondongtang in animal studies was determined to be 1500 mg/kg. We calculated the dose of human based on the results of animal experiments. Human dose was included in the daily intake range of commercial products (see, discussion section). The important facts in this manuscript are that anti-asthmatic effect has been demonstrated within the stable dose range of Macmoondongtang, and the action mechanism has also been clearly identified.

liquiritin and glycyrrhizin were designated as the main marker of Macmoondongtang and the test method for content analysis

(see. https://oasis.kiom.re.kr/oasis/pres/prdetailView2.jsp?idx=32&selectname=null&srch_menu_nix=null#view04) was defined in KFDA (Korea Food & Drug Administration). In this study, we confirmed that liquiritin and glycyrrhizin are markers for quality control and one of the effective markers.

b. There is no data supporting the title: “Macmoondongtang modulates asthma-related changes via modulation of TNF-a andTbet& IFN-g in an asthma murine model”. They showed correlation, nut not causality.

Ans) Thank you so much for your comments and I agreed with your comments. I think that the manuscript title should be changed to “Macmoondongtang controls the asthmatic changes via modulation of Th1-/Th2-/Th17-related factors in an asthma murine model”

3. The reviewer also concerned about the quality of some data. For example, fig 5, it is hard to tell the localization of the transcriptional factors (Tbet and GATA3) at the current magnification. Fig 6, 7, 8, it would be nice to quantify the IHC signals.

Ans) Thank you so much for your comments. We focused the localization of specific protein in the lung tissue such as in nucleus or in cytoplasm and I evaluated the localization of them based on the criteria that in dissimilar to general color when the different colors of light exist are summed-up they change the white color. Therefore, in the asthma-induction group we could evaluated that both of T-bet (green color) and GATA-3 (red color) existed in the nucleus (blue color) as in the nucleus the white spots were found compared to the other groups.

4. Methods are missing: RNA isolation, cDNA synthesis, qPCR methods, Elisa for IgE,

Ans) Thank you so much for your comments and I add the description about materials and methods in the Section.

5. Fig 1, please also include the control HPLC data for glycyrrhizin and liquiritin

Ans) Thank you for your kind advise. we modified the figure 1 according to reviewers’ recommendation.

6. For statistical analysis, how many times have these experiments been repeated? It says mean ± SEM in the figure legends but mean ± SD in the Materias and Methods, which one is correct?

Ans) Thank you so much for your comment and there were typos. I amended the expression as “mean ± SD”.
